# Supplementary material for: Bayesian latent class models for identifying canine visceral leishmaniosis using diagnostic tests in the absence of a gold standard
Source: PLoS Negl Trop Dis. 2022 Mar 14;16(3):e0010236. doi: 10.1371/journal.pntd.0010236 (PMC8947804; doi:10.1371/journal.pntd.0010236)
Supplement: Attachment — Submitted filename: Reviewer Responses - Point by Point.docx [file pntd.0010236.s002.docx]

Reviewer's Responses to Questions

Key Review Criteria Required for Acceptance?
As you describe the new analyses required for acceptance, please consider the following:

**Methods**
-Are the objectives of the study clearly articulated with a clear testable hypothesis stated?
-Is the study design appropriate to address the stated objectives?
-Is the population clearly described and appropriate for the hypothesis being tested?
-Is the sample size sufficient to ensure adequate power to address the hypothesis being tested?
-Were correct statistical analysis used to support conclusions?
-Are there concerns about ethical or regulatory requirements being met?

*Reviewer #1*: The authors used the babesyan test model to associate and detect dogs with possible asymptomatic and symptomatic visceral leishmaniasis. The animals were tested by PCR and serological methods. The number of dogs tested is small and the association using this mathematical model was inconclusive, as the authors themselves present in the discussion. The choice of asymptomatic dogs is not clearly described in the study.

Thank you for your comments. We have incorporated a simulation study to evaluate the relationship between sample size and uncertainty in the gamma mixture latent class model. We have included details of the simulation study in Section 3.5 and Section 4.3; Figure 3 shows the change in uncertainty for different combinations of sample size and disease prevalence. The model performs with more certainty for larger sample sizes. We have also added some clarification around asymptomatic dogs to both the introduction and methods sections to indicate that they were excluded from the analysis.

*Reviewer #2*: Yes, objectives of the study are clearly articulated. Study design and statistical analyses are appropriate.

--------------------

**Results**
-Does the analysis presented match the analysis plan?
-Are the results clearly and completely presented?
-Are the figures (Tables, Images) of sufficient quality for clarity?

*Reviewer #1*: The description of the methods is not clear. The methodology of PCR and serological testing should be better and more clearly described. Most importantly are such methods reproducible?

Thank you for your comment regarding the clarity. We have revised the description to make it more understandable. We have also added some references regarding the reproducibility of these methods, which has been well-studied.

*Reviewer #2*: Results are clearly and completely presented. Figures (Tables, Images) are of sufficient quality for clarity.

--------------------

**Conclusions**
-Are the conclusions supported by the data presented?
-Are the limitations of analysis clearly described?
-Do the authors discuss how these data can be helpful to advance our understanding of the topic under study?
-Is public health relevance addressed?

*Reviewer #1*: Sample banks of positive and negative dogs for visceral leishmaniasis were used. I ask what is understood in the text. The limitations of the methodology used are discussed by the authors and are not few. What unfortunately does not prove the fidelity of the study. Anyway, this method does not seem feasible in public health. The detection of asymptomatic dogs is a great challenge, especially in endemic areas of developing countries. Although the authors' attempt was praised, the study did not prove to be a possible and simple methodology to apply in such areas. The ideal would be to find a reliable serological test to determine the possibility of an asymptomatic dog a posteriori to develop disease and transmit it. Finally, the authors did not study whether asymptomatic dogs are likely to be reservoirs.

Thank you for your comment. We have added some clarification about the objectives of the study, and we provide some further comments on the feasibility of methods such as those presented here in public health.

First, the objective of the study was to assess the potential role of DPP reader information in a latent class model to reconcile inconsistencies between diagnostic tests. In addition, this method does not rely on the use of clinical exam data, which is advantageous in settings where it may be feasible to draw blood, but there is insufficient time or other challenges hamper a proper physical exam for an animal. In certain countries where leish is endemic, getting clear and complete physical exam can be very difficult or nearly impossible due to the demeaner of the animal. We have tried to clarify these objectives in the revised manuscript. Furthermore, the statistical models presented are implemented in R, which is an Open Source software, making it easily accessible to public health professionals. All code to fit these models is also included in the Supplemental Materials. R is used regularly in public health applications.

Second, we did not use these methods to detect asymptomatic dogs, which we agree is an important factor for understanding progression of disease, and an interesting area for further study. Biologically for this question, it was important to look at the feasibility of using the DPP reader information to identify symptomatic dogs, specifically due to the complex immunological response in dogs due to leish. We have provided a model that provides more clarification and identification of the different immunological presentations of dogs. This is described in the Results and reflected in Figure 2. We apologize for the confusion around asymptomatic individuals in this manuscript – hopefully the revised manuscript addresses these shortcomings. Also, studying whether asymptomatic dogs are likely to be reservoirs was not an objective of this study.

*Reviewer #2*: Conclusions RE supported by the data presented. Limitations of analysis are clearly described.

--------------------

**Editorial and Data Presentation Modifications?**
Use  this section for editorial suggestions as well as relatively minor modifications of existing data that would enhance clarity. If the only modifications needed are minor and/or editorial, you may wish to recommend “Minor Revision” or “Accept”.

*Reviewer #1*: I suggest further review of the article. Let this be clearer especially in the detection of asymptomatic dogs, especially in the description of the methodology, in the separate use of PCR and the technique used to observe and describe the results. Finally, the clinical follow-up of asymptomatic dogs compared to the results obtained could give greater credibility to the results. We have no follow up on this dog population.

We hope that we have clarified the details around asymptomatic dogs satisfactorily. Since this is not a longitudinal study and the methodology is not intended to study the progression of leishmaniasis, follow up is not relevant for the analysis. Availability of such follow up information could certainly lead to some other interesting modeling developments, but the methods presented here were not dependent on follow up data.

*Reviewer #2*: Mathematical notation needs to be improved:

- In equation (1), the probability of the Bernoulli distribution is denoted by \delta_i. However, in the next line, this is shown as \delta_{ij}. Authors need to clarify and reconcile the notation differences (different indices).

Thank you for pointing out this inconsistency. It has been corrected.

- x_i's and the design matrix are not clearly defined.

Thank you - this has been clarified.

--------------------

**Summary and General Comments**
Use this section to provide overall comments, discuss strengths/weaknesses of the study, novelty, significance, general execution and scholarship. You may also include additional comments for the author, including concerns about dual publication, research ethics, or publication ethics. If requesting major revision, please articulate the new experiments that are needed.

*Reviewer #1*: Interesting study in the search for answers to two canine populations using PCR and serological technique. I suggest the authors complement the study as described above and improve the description of the methodology. It is important to validate a reliable and especially simple method for use in a neglected disease. As it is presented, I do not recommend publication and praise the authors, but I do advise improving it with revision.

Thank you for your review. We value your feedback and hope that we have satisfactorily addressed your concerns.

*Reviewer #2*: Authors discuss an interesting application and successfully take advantage of the Bayesian hierarchical modeling framework to combine diagnostic data from two different tests (PCR and DPP) to inform the true status of the disease presented by the latent process (D).  In doing so they consider two sets of outcomes: dichotomized PCR and DPP, and dichotomized PCR and DPP reader outcomes and show the benefits of using the DPP reader data (as opposed to the common practice of using dichotomized DPP outcomes).

Authors also take advantage of the Bayesian framework by considering informative priors based on sensitivity and specificity of the tests.

Authors do a good job of discussing the limitations of their case study. Mainly, the fact that majority of their cases have negative PCR and DPP outcomes and the number of positive PCR outcomes is very small. Authors may address the limitations of the data and in order to show the merits of their approach by conducting simulation studies however, I do realize that such a simulation is not trivial.

Thank you for your review. We have included a small simulation study to illustrate the performance of the gamma mixture model (Model 2) to demonstrate that the predictive uncertainty decreases with increased sample size, and this behavior prevails across various values of population prevalence. The description of this simulation study is in Section 4.5. Results from these studies are discussed in Section 5.3. Necessary code, etc. to carry out these simulations is included in the Appendix.

Mathematical notation needs to be improved:

- In equation (1), the probability of the Bernoulli distribution is denoted by \delta_i. However, in the next line, this is shown as \delta_{ij}. Authors need to clarify and reconcile the notation differences (different indices).

- x_i's and the design matrix are not clearly defined.

We have reconciled the notational differences and clarified the notation – thank you for pointing this out.
